# Promoting access and trust: cohort and data profiles in BMJ Medicine

**DOI:** 10.1136/bmjmed-2026-003139

**Published:** 2026-09-18

**Authors:** Tom Nolan, Neil Martin Davies, Katie Harron, Nazrul Islam, Sophie Cook

**Affiliations:** 1 The BMJ, London, UK; 2 Division of Psychiatry and Department of Statistical Science, UCL, London, UK; 3 K G Jebsen Centre for Genetic Epidemiology, Norwegian University of Science and Technology, Trondheim, Trøndelag, Norway; 4 UCL Great Ormond Street Institute of Child Health Population Policy and Practice, University College London, London, England, UK; 5 Primary Care Research Centre, School of Primary Care, Population Sciences and Medical Education, Faculty of Medicine, University of Southampton, Southampton, England, UK

**Keywords:** Epidemiology, Research design

The new cohort and data profiles series in BMJ Medicine will publish profiles from cohorts, data resources, and consortiums, with an emphasis on data trustworthiness and accessibility

Cohort studies and data resources collect an unprecedented amount of data about human health and social care. These resources open up a world of opportunities in epidemiology and population health to examine the distribution and causes of disease in previously unexplored depth. Recognising the potential role for journals to help researchers navigate the increasingly complex data landscape, we are launching a new series in BMJ Medicine. These cohort and data profile articles will help researchers identify reliable, trusted, and accessible sources of data for their research. The series launches with with three profiles: the UK Household Longitudinal Study, and updates to the Millennium Cohort Study and Mexico City Prospective Study.^1–3^

## The challenge of scale

A clear change in epidemiology since modern cohort studies began, about 75 years ago, is scale. The National Survey of Health and Development (NSHD) collected data from all 16 695 births in England, Wales, and Scotland in March 1946.^4^ This impressive feat in post war Britain is eclipsed by most modern data; large cohort studies,^5^ larger consortiums wherein data from multiple cohort studies are combined,^6^ and even larger data resources that can include data from millions of people (or even an entire population) are now available.^7–9^

However, for epidemiological investigation, size (or scale) is not everything. More important is the appropriateness of the data to answer the specific research question, the quality and the coverage (or completeness) of the data, and the breadth of other potentially critical data (eg, socioeconomic characteristics, household and familial data linkage, and genomic data, to name a few). Cohort and data profiles help surface these elements and provide information in a succinct format that can support researchers, reviewers, clinicians, patients, and the public to determine whether the data are appropriate to answer their research question.

## Trust

Health and social care data give personal value to individuals and a financial value to commercial entities.^10^ The importance of these data to the public is arguably the greatest: when used responsibly, these data can improve lives at a population level. Use of this information requires a social contract and trust between the public and scientists.

Recent news headlines that described instances of data from cohort participants being advertised for sale online^11^ and inadvertently being made publicly available^12^ highlight the importance of trust. Society can only benefit from the wealth of information in these rich datasets if scientists and researchers demonstrate that they can be trusted to safeguard citizens' data. Clear and strict governance around access to data are imperative. Crucially, all those involved with using these datasets, from study leaders, researchers, and students, must take collective responsibility for safeguarding these valuable data.

## Accessibility

Another key challenge is ensuring data is accessible. Valuing healthcare data as highly as we value public goods may help; society benefits from electricity and road networks, sewage systems, and high speed broadband, but each requires expertise, resources, investment, and prioritisation to ensure that they work for the public good. Use of healthcare data is the same, but is only useful to society when scientists can analyse it to make new discoveries.

To be useful, therefore, data needs to be FAIR: findable, accessible, interoperable, and reusable.^13^ The FAIR framework gives scientists working on cohort profiles, data resource profiles, and consortiums a blueprint for building the architecture for successful data sharing.

## A new article series for BMJ Medicine

The new cohort and data profile series in BMJ Medicine aims to support these interconnected themes: helping the academic community to make full use of population data by publishing high quality profiles of leading cohorts, data resources, and consortiums. To meet our standards for publication, an article will need to describe a dataset that is accessible to other researchers, and follow a reporting template describing a core set of characteristics. Authors of BMJ Medicine cohort and data profiles are encouraged to describe how readers can obtain and use their datasets, and explain any barriers to accessibility and interoperability.

Each manuscript is subject to our peer review processes but is additionally assessed by a clinical editor and one or more of our expert series advisers, providing input based on their range of methodological, statistical, and editorial experience, aiming to maintain a high standard of quality, consistency, and readability. Through making these articles open access, they are widely available; clinicians and patients can also learn more about the data underpinning the research they are using to guide clinical care.

These articles are commissioned. We welcome enquiries from authors for cohort profiles, data profiles, and consortium profiles, including profile updates, through our author page on the BMJ Medicine website (https://bmjmedicine.bmj.com/pages/authors).
